# Identification of molecular and physiological responses to chronic environmental challenge in an invasive species: the Pacific oyster, *Crassostrea gigas*

**DOI:** 10.1002/ece3.719

**Published:** 2013-08-12

**Authors:** Melody S Clark, Michael A S Thorne, Ana Amaral, Florbela Vieira, Frederico M Batista, João Reis, Deborah M Power

**Affiliations:** 1 British Antarctic Survey, Natural Environment Research CouncilHigh Cross, Madingley Road, Cambridge, CB3 0ET, U.K; 2 Center of Marine sciences (CCMAR), Universidade do Algarve, Campus de Gambelas8005-139, Faro, Portugal; 3 Instituto Português do Mar e da Atmosfera (IPMA), Estação Experimental de Moluscicultura de TaviraVale Caranguejo, 880, Tavira, Portugal

**Keywords:** Candidate genes, condition index, energetic trade-offs, mTOR pathway, transcriptional profiling

## Abstract

Understanding the environmental responses of an invasive species is critical in predicting how ecosystem composition may be transformed in the future, especially under climate change. In this study, *Crassostrea gigas*, a species well adapted to the highly variable intertidal environment, was exposed to the chronic environmental challenges of temperature (19 and 24°C) and pH (ambient seawater and a reduction of 0.4 pH units) in an extended 3-month laboratory-based study. Physiological parameters were measured (condition index, shell growth, respiration, excretion rates, O:N ratios, and ability to repair shell damage) alongside molecular analyses. Temperature was by far the most important stressor, as demonstrated by reduced condition indexes and shell growth at 24°C, with relatively little effect detected for pH. Transcriptional profiling using candidate genes and SOLiD sequencing of mantle tissue revealed that classical “stress” genes, previously reported to be upregulated under acute temperature challenges, were not significantly expressed in any of the treatments, emphasizing the different response between acute and longer term chronic stress. The transcriptional profiling also elaborated on the cellular responses underpinning the physiological results, including the identification of the PI3K/AKT/mTOR pathway as a potentially novel marker for chronic environmental challenge. This study represents a first attempt to understand the energetic consequences of cumulative thermal stress on the intertidal *C. gigas* which could significantly impact on coastal ecosystem biodiversity and function in the future.

## Introduction

Understanding species' responses to environmental perturbation is critical in predicting climate change effects on biodiversity and consequently, ecosystem functioning. Within any ecosystem, this comprises two major aspects: the impact of change on the endemic species and the potential for colonization by invasive species. The latter have caused dramatic changes in ecological systems worldwide and are major threats to biodiversity (Gurevitch and Padilla 2004; Zerebecki and Sorte 2011). One of the best known European marine invasives, the Pacific oyster *Crassostrea gigas* was introduced in the late 1960s from Asia. Initially, *C. gigas* was introduced to replace the Portuguese oyster *Crassostrea angulata,* which was seriously affected by mass mortality events (Grizel and Heral 1991), but it now has a wide geographic range from Norway to the Mediterranean Sea. While *C. gigas* is a numerically successful invasive, it is also an ecosystem engineer; modifying habitats by constructing large three-dimensional reef structures and inducing major changes in invaded ecosystems (Troost 2010). These structures can dominate intertidal zones and in some instances increase both habitat heterogeneity and ecosystem species diversity, providing shelter for the inhabitants against extreme environmental conditions, acting as a buffer to the environment (Troost 2010).

Successful invasive species generally share certain characteristics that allow them to colonize, establish, and expand their range. However, in the case of *C. gigas,* its spread has been facilitated by aquaculture practices, as farms are generally located in open waters (i.e., lagoons and estuaries) without containment measures, and oysters may spawn several times before harvesting. Also *C. gigas* outcompetes the native species when it is present in a mixed oyster population (Troost 2010). In both laboratory and field sampling, *C. gigas* grows more quickly, has a higher metabolic efficiency, scope for growth, and faster feeding and absorption rates than other closely related species, such as *Crassostrea virginica, C. angulata,* and *Saccostrea glomerata*. Mortality of *C. gigas* is also lower and it has a higher resistance to environmental factors such as turbidity and temperature variation (Bayne 2000; Soletchnik et al. 2002).

The high-temperature tolerance of *C. gigas* explains its wide geographic range and it is found in Russia (ca. 48°N) to the east coast of China (30°N). In Europe, *C. gigas* can reproduce in the Danish Wadden Sea (60°N, Wrange et al. 2010) if the water temperature rises above 16–18°C long enough for spawning and larval development and in warmer waters as far south as 37°N in southern Portugal. *C. gigas* withstands short-term exposure to temperatures close to freezing point and up to 35°C and its tolerance to a broad pH range explains its ability to colonize environments such as estuaries that have large natural fluctuations in pH and salinity (Yaroslavtseva et al. 1990). Few studies have examined the physiological and molecular mechanisms responsible for tolerance to wide ranges of environmental conditions in invasive species. Therefore, laboratory manipulation can be extremely useful in determining biological signatures to specific environmental challenges and also the interaction between different types of challenge. In *C. gigas*, these studies have concentrated on understanding the causes of devastating mass summer mortality events, which appears to result from a complex interaction between environmental conditions and disease susceptibility (Li et al. 2007; Chaney and Gracey 2011), but more recently on the effects of ocean acidification (Beniash et al. 2010; Lannig et al. 2010; Tomanek et al. 2011). However, although these latter experiments recorded detrimental effects for both metabolism and shell production of *C. gigas* they were very short term, ranging from a few days to a maximum of a month. Similarly studies on the impact of other environmental stressors, such as heat and hypoxia, have also been carried out for less than 1 month (Meistertzheim et al. 2007; Sussarellu et al. 2010). While short-term experiments can provide insight into stress pathways (Tomanek et al. 2011), they represent acute responses. Short- and longer term exposures can produce very different outcomes when gene expression profiles are measured (Clark and Peck 2009a). Therefore, acute responses may not accurately reflect the long-term constraints of life in new conditions or the subtle trade-offs in energy balance and physiology associated with acclimation to a permanent gradual shift in environmental conditions, such as the predicted increases in sea surface temperature (SST) over the coming decades. This could particularly impact on intertidal and shallow subtidal species and lead to altered seasonality and reproduction patterns, as these species have been argued to be especially vulnerable to climate change because they are close to their physiological limits already (Somero 2010).

Gene expression analyses can be effective in identifying key processes underpinning acclimatization and were used in many of the studies of environmental challenge cited here. However, the approach was varied and ranged from analyses of a single gene (Beniash et al. 2010) to the use of microarrays with several thousand genes (Lang et al. 2009; Sussarellu et al. 2010). The latter exploited the clones from a European-developed transcriptomic resource of 29,745 unique *C. gigas* expressed sequence tags (ESTs) (Fleury et al. 2009). These also provide a backbone for short-read transcriptional profiling enabling discovery-led gene expression analyses, as opposed to an a priori candidate gene approach or the clone number constraints of microarrays.

This study represents a multidisciplinary approach to dissect the effects of temperature and pH on physiological parameters and gene expression of an invasive species, *C. gigas* in an extended 3-month study over this species' productive summer period. Physiological parameters (condition index, shell growth, shell repair, respiration, excretion rates, and O:N ratios) were analyzed alongside molecular analyses, using both a candidate gene approach via Q-polymerase chain reaction (PCR) and transcriptional profiling using SOLiD technology with the aim of providing a better understanding of the constraining environmental factors of this invasive under future climate change scenarios, in particular focussing on the ability of the oysters to maintain their shell in the face of decreasing water pH values.

## Materials and Methods

**Figure 1:**
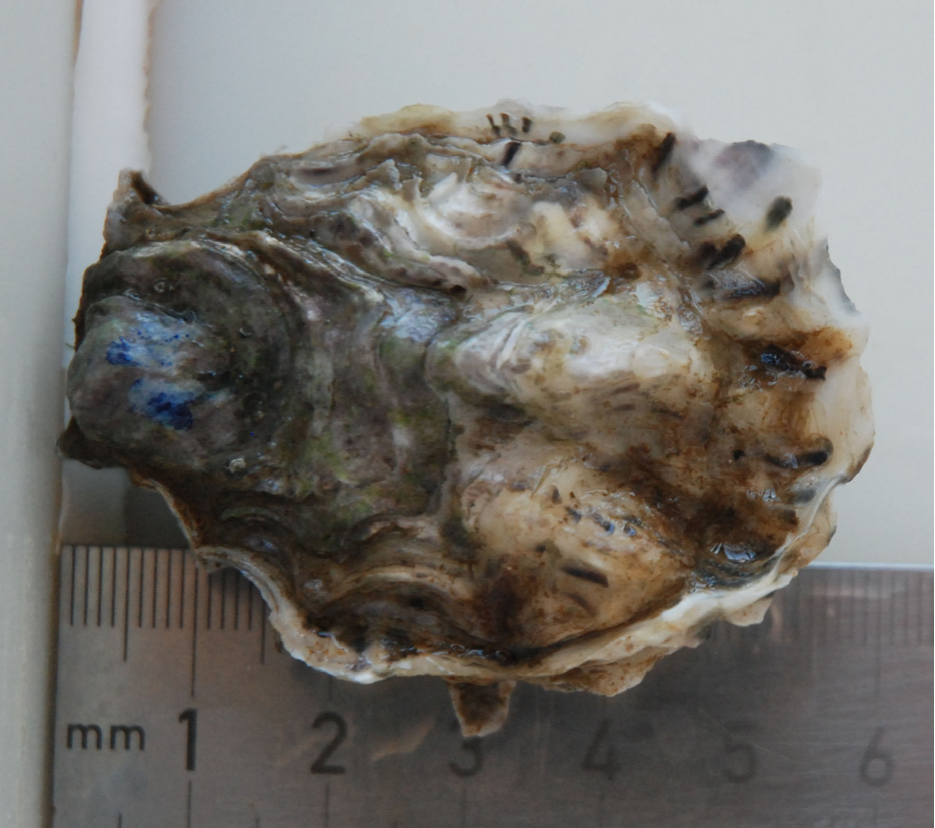
Picture of *Crassostrea gigas*.

Experiments were conducted in the experimental station of CCMAR in the Ria Formosa (36°59'33″N 7°54'17″W, Portugal). The experimental temperatures were chosen to reflect summer temperatures (24°C) and spring/autumn conditions (19°C). The facilities are licensed for animal experimentation and the experiments are covered by a Group-1 license (Direcção-Geral de Veterinária, Portugal). Animals from the same cohort were obtained from collectors placed in farmed oyster regions in Arcachon Bay in SW France (size <20 g). The oysters (Fig. 1) were then cultivated in ground plots for 1 year in the Ria Formosa (Fuzeta), prior to the start of these experiments.

### Culture system

The flow-through culture system consisted of four tanks with the following temperature and pH conditions: tank 1: normal pH and 24°C (NH); tank 2: low pH and 24°C (PHH); tank 3: low pH and 19°C (PHL); and tank 4: normal pH and 19°C (NL), with low pH being a reduction of 0.4 pH units relative to the untreated seawater obtained from the Ria. The seawater was supplied from the Ria Formosa, which was pretreated through both sand and 20-μm filters. Each temperature circuit had a 250-L header tank and two experimental 50-L tanks, one for each pH treatment. Each tank was continuously aerated, with a water inflow rate resulting in a total water exchange three to four times per day. A 12 h light/12 h dark photoperiod was used and pH was modified via CO_2_ injection controlled by an Aqua Medic – pH computer set and a solenoid valve. The temperatures in the experimental and header tanks were recorded every 30 min using a Maxim–Dallas data logger (DS1920L). The output from both CO_2_ and temperature monitoring sensors was used in real time to modify pH and/or temperature. Salinity was measured with a VWR EC 300 – conductivity meter; and pH was measured with an OxyGuard Handy pH meter (Table 1). The oysters were fed a mixture of the microalgae *Isochrysis galbana* (clone T-ISO) and *Tetraselmis suecica* in a proportion of 1:1 in terms of biomass, supplied in continuous flow at a standard concentration of 18,000 cells/mL. The food input in each tank was monitored via the concentration of chlorophyll-a (Chl-a), using a portable fluorometer (10 AU-Turner Designs, Turner Designs, Sunnyvale, CA). Given the complexity of the experimental system and the facilities available, it was not possible to construct three completely separate circuits as true replicates. A total of 30 animals were put into each tank, with different sets of individuals used for the morphometrics, the shell damage repair experiment, the metabolic measurements (oxygen consumption, nitrogen excretion, and O:N ratios), and the transcriptional profiling (Q-PCR and SOLiD analyses) to explore the full range of variability within the experimental populations.

**Table 1:** Water parameters over the course of the 3 month experiment in each of the treatment tanks and mortalities

	Tank 1 (NH)	Tank 2 (PHH)	Tank 3 (PHL)	Tank4 (NL)
	24°C, normal pH	24°C, low pH	19°C, low pH	19°C, normal pH
Temperature	24.3 ± 0.7	24.2 ± 0.3	18.8 ± 0.1	19.4 ± 0.5
pH	7.89 ± 0.01	7.45 ± 0.02	7.46 ± 0.02	7.84 ± 0.02
Salinity (%º)	35.9 ± 0.3	35.9 ± 0.3	36.2 ± 0.1	35.5 ± 0.5
Oxygen (%)	88.2 ± 2.8	88.3 ± 2.6	82.9 ± 4.9	87.9 ± 3.2
Oxygen (mg/L)	6.25 ± 0.37	6.54 ± 0.07	7.15 ± 0.05	7.17 ± 0.05
Mortalities (%)	37.9	6.6	10	3.3

### Morphometrics, shell damage repair, and condition index

The specimens with a mean wet weight of 28.1 g (±0.6 g standard error [SE] mean) were photographed before they were introduced into the experimental circuit and at the end of the experiment. Shells were numbered to facilitate matching of individual oysters during the experiment. Six oysters from each treatment were sampled after 3 months. Tissue and shell wet weight were measured for each animal. Small pieces of mantle tissue were flash frozen in liquid nitrogen for molecular analyses. Tissues and shells were dried at 60°C for 2 days and the dry weights (DW) of each noted. The condition index was calculated according to Davenport and Chen (1987), where CI = (dry tissue weight/dry shell weight) × 100. A further six oysters from each treatment had a small hole drilled in the shell using a standard household drill and were replaced in the system for a week and then assessed for their ability to repair this breach in their external structure. For measurements of shell repair after drilling, photographs were taken of each of the holes and the area occluded measured using ImageJ (http://rsb.info.nih.gov/ij/).

### Metabolic measurements (VO_2_)

Individual animals were placed in sealed respirometers containing seawater saturated with oxygen. The oxygen (O_2_) concentration was measured at 30, 60, and 90 min, until it fell by 30%. Chambers without animals were used as controls. Oxygen concentration was measured with a YSI 5730 dissolved oxygen electrode connected to a YSI 58 BOD meter. Respiration rate was calculated from the difference in O_2_ concentration between the chambers with and without animals, using the following expression:


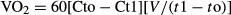


where Cto and Ct1 were the dissolved oxygen concentration (mL O_2_/L) at the start and end time (minutes) of the experiment, respectively, *V* was the volume of the respirometer, and *t*1 − *t*o represented the start and end times (minutes) of the measured period, respectively.

### Ammonia excretion measurements

Oysters were placed in glass containers filled with 250 cm^3^ seawater previously filtered through 0.2-μm Millipore membranes. After 90 min, samples of 10 cm^3^ were collected from each chamber and frozen at −20°C. The ammonia excretion rate was determined according to the phenol-hypochlorite method (Solorzano 1969) using the following formula: μgNH_4_–N/h = [(μmol/L test – μmol/L control)·(14/(1000/*V*))·(1/*t*)], where *V* was the volume of the experimental container (0.25 L); *T* was the incubation time (1.5 h), and μgNH_4_–N was the concentration of ammonia–N (μmol/L).

### O:N ratio index

The metabolic rate measurements were calculated in mL O_2_/h and the ammonia excretion results in μmol/L NH_4_/h. To enable the ratio of oxygen consumed to nitrogen excreted (O:N index) to be calculated, these data were converted into the same units (mg atoms) according to Widdows (1985).

### Size standardization

Physiological rates were corrected to a standardized weight of an individual to preclude variability in the physiological rates engendered by size differences. When physiological measurements were completed, the shell length of each individual was recorded to the nearest 0.1 mm and the soft tissues excised from the shell, dried at 110°C for 12 h and weighed for DW determination. Metabolic and excretion rates were obtained using the following formula: Ys = Ye × (CPst/CPe)*b*, where Ys and Ye were the corrected and noncorrected physiological rates, respectively; CPe was the DW of the individual clams (mg); CPst was the corporal parameter standard 1000 mg DW and *b* was the power value that scaled physiological rate to body weight using *b* = 0.75 (Bayne and Newell 1983) for the respiration rate and *b* = 0.72 (Hawkins et al. 1985) for the excretion rate.

### Statistical analyses on morphometric data and biochemical analyses

Statistical analyses were performed in Minitab v15 (Minitab Inc., State College, PA). If preliminary screens of the data showed normality of distribution (Bartlett test) and homogeneity of variance, then analyses of variance (ANOVA) (general linear model) with post hoc Tukey test analyses was used. If tests for normality and homogeneity of variance revealed non-normality of the data set or heterogeneity of variance, even after transformations, then nonparametric Kruskal–Wallis tests were used.

### Molecular analyses

All molecular analyses were carried out using the mantle tissue. This is one of the biggest organs in oysters and has multiple functions including shell formation, secretion of the ligament, sensorial activities, respiration, and storage (i.e., glycogen). A number of transcriptome studies have been published for this organ, enhancing the annotation potential of transcripts (Clark et al. 2010; Jackson et al. 2010; Kinoshita et al. 2011). Additionally, gene chip studies have shown that bivalve tissues directly in contact with the external environment are more responsive to environmental perturbation than internal tissues (Clark et al. 2013; plus unpublished data). Hence, transcription profiles of the mantle can act as an effective proxy of whole-animal response, but this is also the organ that secretes the shell and therefore most likely to show the effects of a compromised ability to mobilize calcium (under reduced pH, e.g.). The aim of many environmental molecular studies is to use laboratory experiments to identify “stress” biomarkers and transfer their use to the natural environment for early warning systems (Truebano et al. 2010). Initially, the scope of the molecular analyses was to study the transcription patterns of a panel of candidate genes in mantle tissue, but, subsequently, we were able to extent these analyses to more extensive transcriptional profiling.

### RNA extraction and reverse transcription

Total RNA was extracted from mantle tissue using the Maxwell® 16 Total RNA Purification Kit (Promega, Madison, WI) according to manufacturer's instructions. Samples were then treated with a DNA-free Kit (Ambion, Austin, TX) to remove genomic DNA, and reverse transcription was carried out using 500 ng of total RNA, random hexamers, and MMLV reverse transcriptase (Promega) according to manufacturer's protocols.

### Q-PCR primer design and analysis

Primers were designed using Primer3 software (http://frodo.wi.mit.edu/). Primer pairs that gave a single product with the expected size and absence of primer dimers (Table S1) were then tested for real-time PCR. The Q-PCR assays were performed in duplicate in a total volume of 15 μL (7.5 μL of 2× SYBR® Green supermix [Biorad, Hercules, CA], 1 μmol/L of each primer, and 2 μL of cDNA [1:5 dilution]). For all genes, PCR cycling conditions were as follows: 10 min at 95°C, followed by 45 cycles of 15 sec at 95°C, and 1 min at 60°C. A final melt curve was carried out between 60 and 95°C. Each run included cDNA samples and blank no template controls. PCR efficiency (*E*) was determined using standard curves from a serial dilution of pooled cDNA from all treatments for each primer pair. Baseline corrections and *C*_t_ value determination were performed using StepOne software v2.0 (Applied Biosystems, Foster City, CA). Relative quantification was done by normalizing raw *C*_t_ values to the reference gene using the formula, *E*_target_ (*C*_t_ target)/*E*_Reference_ (*C*_t_ reference). No significant differences (Kruskal–Wallis test, *P* > 0.05) in *C*_t_ values and a low coefficient of variation (4.3%) were observed for the actin gene among treatments, justifying its use as the reference gene. Differences in the relative expression of each gene among treatments were analyzed by one-way analysis of variance (ANOVA).

#### Transcription profiling

RNA was isolated as described above. Five individuals from each treatment were pooled and subjected to SOLiD transcriptional profiling at the Natural Environment Research Council Biomolecular Analysis Facility at Liverpool. A total of 205,888 *C. gigas* ESTs from the public database were assembled using CAP3 with default parameters to construct 20,125 contigs (Table S2), which were then used as the reference for mapping the SOLiD sequences. This consisted of the following groups of paired-end reads: 45,900,175 (NH), 36,811,031 (PHH), 38,477,719 (NL), and 41,668,977 (PHL). The paired reads were mapped to the contigs with Maq (Li et al. 2008), using default parameters. Expression analysis was carried out on all the pairwise combinations, rather than a factorial design (Table 2). Filtering consisted of removing mappings in which one of the treatments had no mapping for a given contig. Normalization was carried out by dividing counts by library size. Selection for differential expression consisted of two approaches for added stringency: a twofold expression-level difference, and the use of a linear model in Bayseq (Hardcastle and Kelly 2010), with a Benjamin–Hochberg adjustment for multiple testing (Benjamini and Hochberg 1995) with a cutoff set at 0.01. For the linear model, a proxy replication for mapping variance consisted of the separate mappings of the paired-end reads to the contigs. Only mappings where both paired-end reads mapped to the same contig were used to generate expression levels and calculate significance of expression (Table S2). Contigs were searched for sequence similarity using Blast (Altschul et al. 1997) against the GenBank nonredundant database (Benson et al. 2007) with a threshold score of <1e^−10^. All annotations were manually verified. All sequence data were submitted to the NCBI Short-Read Archive (SRA) with the accession number: SRA064041.

**Table 2:** Mapping comparisons carried out on the SOLiD data, detailing the number of up-regulated clones for each comparison

			Upregulated clones	Upregulated clones
Comparison 1	NH vs. NL	Effect of temperature under normal pH	NH 66 (28)	NL 25 (8)
Comparison 2	PHH vs. PHL	Effect of temperature under lowered pH	PHH 41 (15)	PHL 105 (36)
Comparison 3	NH vs. PHH	Effect of pH under summer conditions	NH 81 (35)	PHH 33 (14)
Comparison 4	NL vs. PHL	Effect of pH under spring conditions	NL 9 (3)	PHL 74 (22)

Figures in brackets denote the number of clones with putative annotation using Blast sequence similarity searching. Treatment codes: NH: 24^o^C, normal pH; PHH: 24^o^C low pH; PHL: 19^o^C low pH; NL: 19^o^C normal pH.

## Results and Discussion

All the oysters used in this study came from the same year cohort and were held under identical conditions prior to the start of the experiment, with a year's cultivation in the Ria, prior to the start of this experiment, and hence were of a similar size, age, and physiological condition. The temperatures used in this study were well within the normal temperature ranges of the Ria Formosa lagoon. Annually the temperatures vary from 12°C in winter to 27°C in summer (Newton and Mudge 2003). So in the study described here, the 19°C treatment corresponded to an average spring/autumn temperature, whereas 24°C represented a high temperature that regularly occurs in the summer. The aquarium system was stocked with water collected directly from the Ria Formosa. This water source has a naturally lower pH, compared with open ocean waters, so ambient water pH for the experiment was approximately 7.9 with the experimentally lowered pH being reduced by 0.4 pH units. Water was taken from the Ria at high tides only, from the main channel, where there is no stratification (Newton and Mudge 2003) and hence salinity remained constant throughout the period of the experiment (Table 1).

### Mortality and spawning

There was some mortality in each of the tanks during the 3-month culture (Table 1) ranging from 3.3% in the 19°C ambient pH tank to almost 38% in the 24°C ambient pH tank. One further animal died in the 24°C ambient pH group after the shell had been drilled, presumably as a result of trauma during the drilling process. High mortalities are often observed in *C. gigas* at high temperatures (Perdue et al. 1981), but mortalities in the 24°C, low-pH tank were only 6.6% (Table 1). Given that in each temperature circuit both tanks were fed from the same header tank, that is, the water supply in the 24°C ambient and 24°C low pH was the same, it was unlikely that disease was the cause. The animals in the 24°C ambient pH tank spawned first and the losses occurred directly after this spawning event, which may explain the loss of physiological condition of the oysters and/or water quality. Gamete release was probably less intense in the other treatments, as both low temperatures and pH delay spawning and decrease gamete quantity (Loosanoff and Davis 1952; Calabrese and Davis 1966).

### Condition index

**Figure 2:**
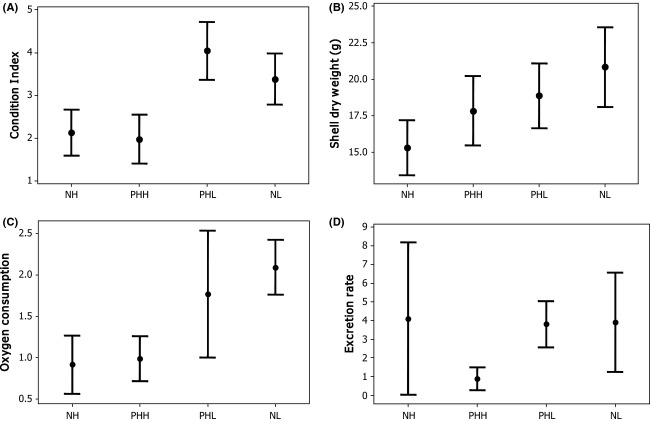
95% confidence intervals for mean values based on pooled standard deviation of (A) condition index; (B) shell dry weight (g); (C) oxygen consumption rates (mL/O_2_/h, corrected to a standardized weight of an individual); and (D) excretion rates (NH_4_ μmol/L/dry weights [g]/individual/h, corrected to a standardized weight of an individual). *X*-axis symbols: NH: 24°C, normal pH; PHH: 24°C low pH; PHL: 19°C low pH; NL: 19°C normal pH.

Condition index (CI) reflects the physiological condition of the animals due to their activity (e.g., reproduction, growth, health status) under certain environmental conditions. It also reflects the energy balance which can vary considerably in filter-feeding organisms in the natural environment depending on phytoplankton availability and the physiological requirements of the animals (Bayne et al. 1976; Norkko and Thrush 2006). Significant differences were detected in the CI results between treatments; GLM showed an effect of temperature: *F*_1,43_: 38.35, *P* < 0.001, but no effect of pH (*P* = 0.349) on CI and no interaction between temperature and pH (*P* = 0.137). Hence, animals kept at 19°C showed a significantly higher tissue to shell mass ratio, irrespective of pH (Fig. 2A). In this experiment, animals were fed ad libitum, at higher levels than the Mediterranean bloom and also above that required to produce maximum clearance rates in grazing experiments (Dupuy et al. 2000). Differences in CI were thus purely due to treatment effects (Fig. 2A). As the CI was not taken at the start of the experiment, the lower CI at 24°C could be due to either a net loss (via mobilization of stored reserves) or a failure to accumulate reserves as fast as the animals at 19°C.

### Shell growth and damage repair

Because of the irregular shape of cupped oysters, dry shell weights were used as the metric of growth. The GLM analyses showed a significant effect of temperature on dry shell weights (*F*_1,43_ *P* = 0.003), but no effect of pH (*F*_1,43_ *P* = 0.791), although a significant interaction occurred between temperature and pH: *F*_1,43_ *P* = 0.037 (Fig. 2B). The post hoc Tukey test showed that animals kept at normal pH at 19°C had significantly (*T* = 3.751, *P* = 0.003) heavier shells than those of animals kept in normal pH at 24°C (Fig. 2B). In the damage repair experiment, a one-way ANOVA of the area occluded showed no significant differences between any of the treatments (*F*_1,20_ = 0.64, *P* = 0.601) (data not shown), indicating that shells were repaired at similar levels, irrespective of treatment. The fact that animals at the higher temperatures had lighter shells (Fig. 2B) indicated that they had reallocated energy away from growth. However, the shell growth results contrast with those from the damage repair experiment. The lack of difference in damage repair capacities under the different treatments indicated that the requirement to repair a breach in the external defenses took priority over other processes.

### Oxygen consumption, excretion rates, and O:N ratios

Metabolic rate data were non-normally distributed, so a nonparametric Kruskal–Wallis analysis was used and showed a significant effect of temperature (*P* < 0.001), but no effect of pH (*P* = 0.404) (Fig. 2C). Although a large variability in excretion rates was measured within treatments (Fig. 2D), there was a significant effect of temperature (*P* < 0.007), but no effect of pH (*P* = 0.100) (Fig. 2D). The large variability in excretion rates was potentially confounded by lower numbers of animals sampled from the 24°C normal seawater tank (*n* = 5, with *n* = 10 in all other treatments) and four animals in the 24°C low-pH tank, where the excretion rates were not detectable above background levels. The O:N ratios indicated that there was a difference in fuel substrate utilization among treatments. The 19°C oysters had similar O:N ratios of 55.8 (PHL) and 68.8 (NL) and thus were burning mainly carbohydrates and lipids with a small amount of protein, a similar result to those measured seasonally in the natural environment (Mao et al. 2006). At 24°C, oysters in normal seawater were burning an increased amount of protein with an O:N ratio of 26.7. The reduced ammonia production in the PHH oysters resulted in a higher ratio of 137.6, which indicated that they had burnt all their available reserves of protein and were predominantly metabolizing carbohydrates and lipids, potentially from any gonad material remaining after the earlier spawning events. Given the lower CI of the animals at 24°C, it was surprising that their oxygen consumption was lower than the 19°C animals (Fig. 2C), particularly as biochemical reaction rates approximately double with every 10°C rise in temperature (the *Q*_10_ coefficient). Two factors may have contributed to this: First, the 19°C animals were close to a second spawning event, which likely increased metabolic rate. Of more importance may be that the 24°C oysters were unable to consume enough food to fuel their increased metabolic requirements, as evidenced by their lower CI and reduced shell growth (Fig. 2A–D). The reduction in both metabolic and excretion rates in these animals indicated that they were likely reducing metabolic rates to conserve energy, a strategy often adopted by animals in stressful environmental conditions (Hand and Hardewig 1996).

### Molecular analyses

#### Q-PCR

**Figure 3:**
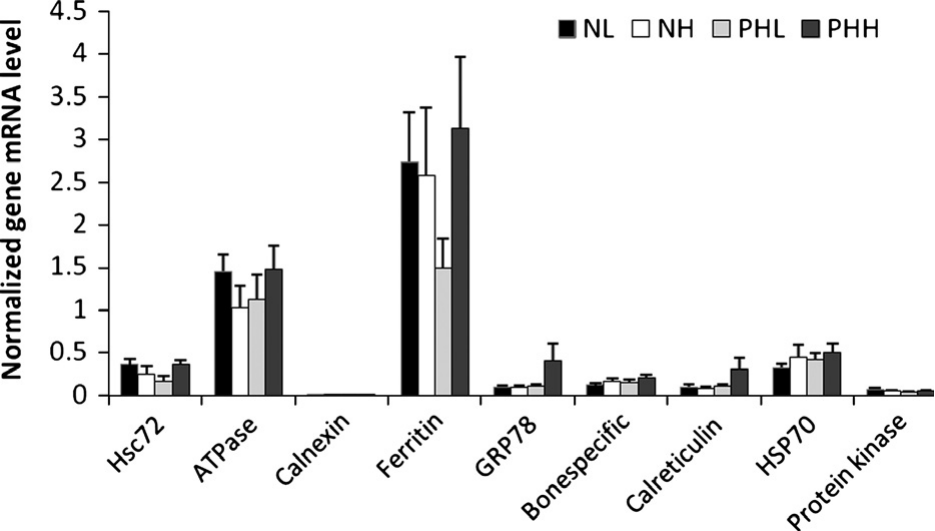
Mean mRNA levels (*n* = 6) of ATPase (P-type sodium–potassium ATPase), Hsp70, Hsc72, bone-specific (similar to bone-specific cmf608), calreticulin, protein kinase (calcium-dependant protein kinase), ferritin, calnexin, and GRP78 in the NL, NH, PHL, and PHH treatments. The error bars represent standard errors. The *Y*-axis represents relative gene expression levels compared with the reference gene.

Initially, the scope of the molecular analyses was to study the transcription patterns of a panel of candidate genes in mantle tissue, targeted at the stress response, shell production, and calcium regulation. These included representatives of the heat-shock protein family and ferritin (an oxidoreductase), which are all upregulated in response to different stresses in molluscs (Zhou et al. 2008; Clark and Peck 2009a). These were supplemented by potential biomarkers for the effect of pH, with a P-type sodium/potassium ATPase, which is implicated in ion membrane transport and several calcium-binding proteins (Table S1). A further sequence was included which was labeled as “bone-specific”, which showed strong sequence similarity to cmf608, a unique marker of early osteochondroprogenitor cells in *Homo sapiens* (Segev et al. 2004); although its function in other species remains unknown, it presents a reasonable candidate for further investigation in lesser characterized nonmodel species. No significant differences (ANOVA, *P* > 0.05) were observed among the four treatments for all genes studied (Fig. 3). These data were validated in the SOLiD transcriptional profiles, as none of the candidate genes used in the Q-PCR was significantly upregulated, although they were present in the mapping data. Some of the previous studies investigating environmental stress in oysters have included Q-PCR analysis of selected candidate genes (Li et al. 2007; Beniash et al. 2010; Dickinson et al. 2012). But as we have shown here, candidates known to undergo elevated expression levels under acute challenges were not necessarily those most expressed under chronic conditions. Indeed, the genes used in the previous studies, including carbonic anhydrase, a gene often used as a biomarker for calcium mobilization (c.f. Beniash et al. 2010; Dickinson et al. 2012), were absent from our upregulated gene lists, but present in the EST backbone, against which the SOLiD data were mapped. Thus, transcriptional profiling was subsequently employed in a gene discovery-led approach.

### SOLiD transcriptional profiling

Transcriptional profiling was undertaken to provide a more detailed picture of the cellular events underpinning the physiological responses of the oysters to the different culture conditions. A specific aim was to dissect out the differences in response to the two environmental variables and hence we applied a simplified analytical framework where one variable was held constant to access the effect of the change in the second variable. A series of four comparisons were carried out, as detailed in Table 2, with the informative genes discussed below, and transcripts per million (TPM) details of all these transcripts are in Table S2.

#### Comparison 1: The effect of temperature under ambient pH conditions

**Figure 4:**
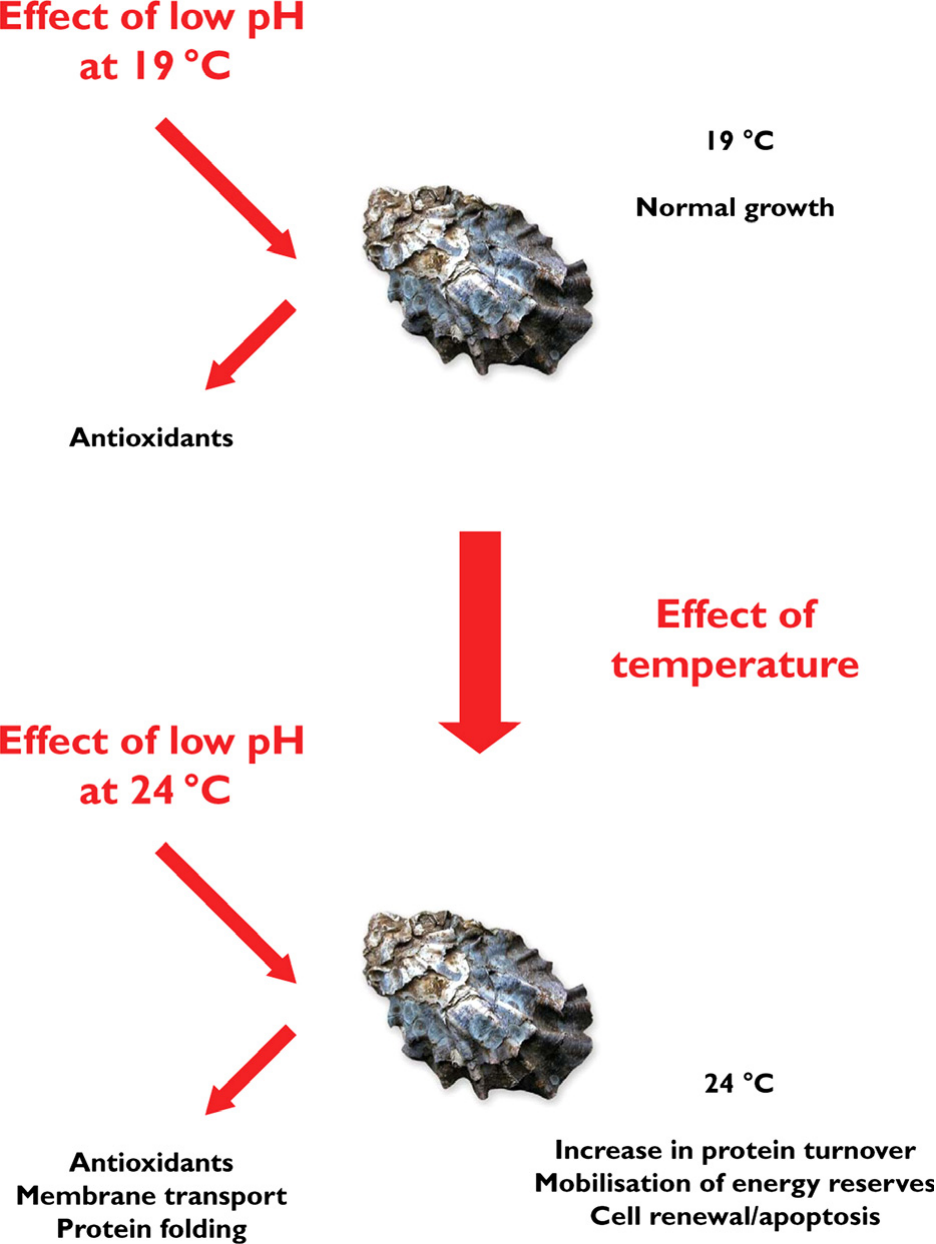
Diagrammatic representation of molecular results, which are fully described in Tables S3–S6.

Transcripts that were upregulated in the animals maintained at 19°C were indicative of normal growth activities (Fig. 4, Table S3) with several transcripts showing sequence similarity to proteins involved in cytoskeleton and muscle development and also a transcript for gigasin, the organic matrix of calcified shell layers in *C. gigas*. This is in agreement with the higher dry meat weight and dry shell weight observed in the animals kept at 19°C. A different transcriptional profile was presented by the animals held at 24°C (Fig. 4, Table S3). As the animals in this experiment had spawned, they would be expected, as part of their normal cycle, to be increasing carbohydrate (Patrick et al. 2006). However, transcripts more indicative of mobilization of stored reserves, such as the homeostatic control of glucose, breakdown of lipids, and movement of lipids between cellular compartments, were upregulated. These included glucose-6-phosphate translocase (Table S3), which is involved in the homeostatic control of glucose and stereogenic acute regulatory protein, propionyl-CoA carboxylase, and nuclear receptor coactivator 4 that are associated with lipid metabolism. The latter has a key regulatory role, as it contains ARA70 domains which interact with the peroxisome proliferator-activated receptor gamma (PPARγ), which plays a key role in lipid metabolism and storage and also activates a number of genes with related functions, including lipoprotein lipase and AP2 (adipocyte fatty acid–binding protein) (Heinlein et al. 1999). The products of both of these transcripts are involved in the breakdown of lipids and as a consequence, release energy for use by the cells. This lipolysis is facilitated by movements of lipids between cellular compartments, as evidenced by transcripts with sequence similarity to members of the ATP-binding cassette and vacuolar protein sorting associated protein families. These data linked back directly to the CI results and the inability of the animals to assimilate sufficient food at 24°C. It has been reported that depending on the physiological condition of *C. gigas*, absorbance efficiency can decrease when the temperature is above 19°C (Lambert et al. 2008). This seems to have been the case of oysters held at 24°C independently of the pH values in our study. These data clearly indicated that the physiological condition of the animals kept at 24°C was compromised, potentially to the level where it was not sustainable long term and this was reflected in significantly reduced body and shell weights.

The results also suggested that there was a tension between cell renewal and apoptosis. Putative orthologues of the raptor gene (regulatory-associated protein of mTOR), which is part of the PI3K/AKT/mTOR pathway, were identified. The raptor protein binds to mTOR as part of the mTORC1 complex and mediates mTOR action (Hara et al. 2002). mTOR is a highly conserved nutrient responsive regulator of cell growth found in all eukaryotes. (Gwinn et al. 2008). It is involved in promoting cell growth and is inactivated under stressful conditions to halt cell-cycle progression to conserve energy and repair damage. When mTOR cannot be inactivated, cells continue through the cell cycle and ultimately undergo apoptosis, hence the mTOR–raptor interaction is a critical mechanism by which eukaryotic cells coordinate the rate of cell growth under different environmental conditions (see also comparison 3). Also identified as upregulated in the 24°C animals were putative homologues of protein kinase C iota type and HSP7012B, both of which have been shown to have antiapoptotic and anti-inflammatory roles in other species.

#### Comparison 2: The effect of temperature under reduced pH conditions

In this comparison, while there were transcripts with similar annotations to those transcripts upregulated in the analyses at ambient pH, there was very little overlap in the actual contig identifiers (Fig. 4, Table S4). This is almost certainly because the short reads mapped to different members of large protein families, such as gigasin and collagen. There were only two clones in common between the 19°C data sets and two between the 24°C data sets. No single contig was present in all the data sets. The 19°C animals cultured at the lower pH showed the expression of transcripts with putative functions in normal cellular functioning and cell growth; in particular, in the presence of gigasin, the shell matrix protein was noted, which again correlates with increased shell production and thickness at lower temperatures (Table S4). There was also some evidence of cellular stress, with upregulation of transcripts with sequence similarity to, and putative functions as antioxidants, involvement in detoxification processes, and thioredoxin, a classical “stress” protein that has been identified in oyster species after bacterial challenge (Martin-Gomez et al. 2012; Xu et al. 2012). At 24°C, a different member of an antioxidant family was present, a putative sigma class glutathione s-transferase, plus two other transcripts for the chaperones protein disulphide isomerase (PDI) and HSP60, which are involved in protein folding and an enhanced requirement for which is often associated with cellular stress. Hence, this comparison revealed potentially subtle differences in cellular stress, as a result of synergistic actions between temperature and pH, but the changes in gene expression did not impact significantly at the level of the whole animal.

#### Comparison 3: The effect of altered pH at 24°C

Animals in both ambient and low pH at 24°C showed transcripts with similarity to structural proteins involved in the normal activation of the cytoskeleton (Fig. 4, Table S5). The main differences were apparent in the transcriptional profiles from animals in ambient seawater at 24°C. Predominant functions were indications of a tension between cell renewal and apoptosis, with the identification of further components of the PI3K/AKT/mTOR pathway (phosphatidylinositol 4,5-bisphosphate 3-kinase catalytic subunit beta isoform [PI3K] and the MET tyrosine kinase receptor) (Fig. 4, Table S5). Additionally, there were several transcripts (annotated as sushi and notch) largely composed of EGF domains, which may be potentially involved in cell attachment. Also showing enhanced expression was several membrane transporters, transcripts implicated in calcium mobilization, and mobilization of energy stores. Of particular note was a putative mitochondrial calcium uniporter (MCU). Mitochondrial calcium homeostasis via the MCU has a key role in the regulation of aerobic metabolism and cell survival (De Stefani et al. 2011). It was noteworthy that carbonic anhydrase, a gene often used as a biomarker for calcium mobilization (c.f. Beniash et al. 2010; Dickinson et al. 2012) was not present in any of our upregulated gene lists. Finally, similar to comparison 1, there was evidence of enhanced mobilization of energy stores with annotations for phosphoenolpyruvate carboxylase (carbohydrate biogenesis); fructose-1 (gluconeogenesis), and pancreatic lipase-related protein (lipid metabolism). This potentially increased energy mobilization in the animals in normal seawater at 24°C may explain the interaction effect observed in the analysis of the shell weight data, where, in spite of similar CIs, these animals had lighter shells than animals in lowered seawater pH at the same temperature. The animals in normal seawater expressed transcripts, which indicated that they may have been more active in their immune defense (Table S5; e.g., ECSIT [evolutionary conserved signaling intermediate in the Toll pathway]). This was the only set of animals with mortalities occurring at the start of the experiment and so represents a selected subset of the population. It may be that whatever caused the mortality event may have sensitized the surviving animals to the prolonged stressful conditions.

#### Comparison 4: The effect of altered pH at 19°C

There was virtually no overlap of the 19°C data sets, to those at 24°C above, with only one unannotated contig in common in the low-pH conditions. This final comparison was largely characterized by an increased immune response and production of antioxidants in the animals cultured in low pH (Fig. 4, Table S6). The best example of this was a match to the antimicrobial peptide, big defensin 1 from *C. gigas*. This gene is presumed to be expressed only in hemocytes. The presence of these transcripts in mantle tissue may be due to hemocyte infiltration via the circulatory system. There were also other transcripts identified with putative defensive roles, including “classic” antioxidants, such as peroxidasin and glutathione peroxidase. These data indicate that either the animals in the lower pH came under enhanced microbial challenge, or were more sensitive under the altered conditions. But, again, these gene expression changes appear to be subtle with regard to their effect on whole animal's physiology responses and morphometrics.

Previous investigations have reported *C. gigas* as sensitive to both elevated temperatures and lowered pH with significant mortalities predicted (Kurihara et al. 2007; Beniash et al. 2010; Lannig et al. 2010; Gazeau et al. 2011; Tomanek et al. 2011; Dineshram et al. 2012; Barros et al. 2013), although this may vary with different populations (Ginger et al. 2013). However, oysters of the genus *Crassostrea* are generally found in intertidal to low subtidal levels in estuaries and lagoons, environments where conditions can change dramatically. For example, *C. gigas* is able to survive in low- (ca. 5 ppt) and in extremely high (ca. 42 ppt)-saline conditions and it also withstands temperatures from close to freezing point up to 35°C. The pH in the open ocean generally varies considerably less than in estuary waters where it can easily drop below 7. For example, during the 1960s when the Tagus estuary (Portugal) had one of the biggest oyster beds in Europe (species: *C. angulata*), pH values at the surface varied between 5.75 and 7.85 with a mean value of 7.22 (Vilela 1975). Hence, oysters of the genus *Crassostrea* that are well adapted to estuarine environments are likely to have a high tolerance to short-term pH and temperature variation. Given the wide range of conditions tolerated by this species in the natural environment, often exceeding those of the laboratory manipulations, and the fact that it is a successful invasive, such marked effects using laboratory-manipulated pH experiments on oysters were surprising and not necessarily in line with the results described here. This experiment held *C. gigas* at elevated temperatures and lowered pH for an extended period of 3 months across the important summer reproductive period. The compromised physiological condition in the 24°C oysters, reflected in significantly reduced tissue and shell weights, indicated that survival at this temperature was potentially not sustainable in the long term. There were similarities between these results shown here and the biochemical processes associated with summer mortality events, where carbohydrate metabolism decreases and lipid synthesis is arrested (Berthelin et al. 2000). A previous study of *C. gigas* indicated that feeding was only inhibited at 25°C with higher temperatures producing an energetic deficiency. Temperatures of 15–25°C were reported as optimal for sustaining increases in dry mass, even under limiting food availability (Bourles et al. 2009). These data would indicate that 24°C was a reasonable proxy for summer exposure. However, in the natural environment, temperature cycling occurs, whereas here, the animals were held at 24°C constantly. Thus, recovery periods at lower temperatures, where reductions in metabolism at the same feeding rate, which enable the build-up of energy reserves and continued growth, were not possible (Almada-Villela et al. 1982). However, if the average temperature of the Ria remains elevated over the summer in the coming years, the recovery possible during diurnal and more longer term (days) temperature cycling will become less effective with potential chronic depletion of energy stores. Indeed, the climate predictions are for increased SST, which will particularly impact intertidal and shallow subtidal species, as they will have to survive longer extended periods at higher temperatures with potential consequences for energy balance and physiological trade-offs (Somero 2010). Ocean productivity will also change, with altered timing of the phytoplankton bloom and consequentially the decoupling of phenological relationships (Edwards and Richardson 2004; Sommer and Lengfellner 2008). This may be particularly critical for filter feeders if these shifts coincide with the energy intensive reproductive period.

The physiological analyses indicate very little effect of pH, except for a potential interactive effect on shell growth. The exclusive upregulation of antioxidants and chaperones in the transcriptional profiles of animals in low pH was thus interesting. These potentially indicate increased energetic requirements, which are clearly sublethal. Given the physiological analyses, they may represent cellular trade-offs, which would require long-term acclimation trials to determine whether they represent permanent transcriptional changes or transitional acclimation processes. Heat-shock proteins are prime candidates to monitor environmental stress due to their high evolutionary sequence conservation and relative ease of cloning in nonmodel species. However, these genes are not always the most effective indicator of organism stress (Iwama et al. 2004; Clark and Peck 2009b), as was indicated here by the Q-PCR experiments (Fig. 3). Chapman et al. (2011), in a microarray and neural network analysis of oysters from different sites, did not find definitive environmental biomarkers. They suggested that transcriptome profiles relating exposure to specific variables were likely context specific, only visible through a discovery-led approach. The microarray study into heat stress in this species also concluded that the response probably results from a complex interaction of cell damage, opportunistic infection, and metabolic exhaustion (Lang et al. 2009) and cannot be described by analysis of one or two biomarkers.

The lesser pH effect demonstrated here agrees with Dickinson et al. (2012), where salinity was the most important single stressor (out of salinity and pH) on juvenile *C. virginica* over an 11-week experiment. Salinity stress produced an increased energy consumption resulting in energetic deficiency, which the animals could not compensate for. They also found an additive pH effect and suggested that in salinity-stressed environments, pH could change the likelihood of survival (Dickinson et al. 2012). Similarly, related studies on *C. virginica* examining biocalcification showed that temperature and salinity mitigated the effect of reduced biocalcification in lowered pH (Waldbusser et al. 2011) and also that temperature had a stronger effect than moderate hypercapnia (Matoo et al. 2013). These agree with the results described here. Matoo et al. (2013) also showed considerable mortality of *C. virginica* at elevated temperatures regardless of exposure to high-CO_2_ conditions. These data emphasize the complexity of understanding a species' response to environmental perturbation, particularly in the face of multiple stressors and also the challenge of extrapolating the results of laboratory experiments to environmental conditions (Waldbusser et al. 2011). However, laboratory experiments can significantly help in the understanding of the cellular mechanisms underlying the environmental whole-animal responses. Many studies of pH effects on marine organisms are still very short term (weeks rather than months or years) and often concentrate on the larval stage.

The 3 months here were not long term, but the expression profiles did not indicate an acute stress response (cf. Meistertzheim et al. 2007; Matoo et al. 2013). In a study of thermal stress in *C. gigas*, the Q-PCR time course of 17 genes showed a graduated response: a high and rapid increase at 3–7 days, a decrease at 14 days, and a less pronounced increase at 17–24 days, with protein synthesis reduced after 24 days (Meistertzheim et al. 2007). Hence, with our experimental timescale, we were clearly beyond this immediate stress response and if this experiment was greatly extended, then very different expression profiles would likely be generated, more representative of a potentially sustained acclimation response, which could impact on future generations (Parker et al. 2012).

As oysters of the genus *Crassostrea* occur intertidally and are adapted to variable environmental conditions they could therefore be expected to thrive under future climate change scenarios, especially, *C. gigas* which outcompetes other *Crassostrea* species in mixed populations and is a coastal invasive (Bayne 2000; Soletchnik et al. 2002; Troost 2010), but clearly this may not be the case. The recent publication of the oyster genome has shown an increased number of “stress” genes in this species (Zhang et al. 2012), which might be expected in a species inhabiting environments where conditions can change rapidly and acute short-term tolerance to environmental challenge is essential for survival. What remains unknown regarding future climate change scenarios is how irreversible changes in one environmental variable affects the interplay and trade-offs between the other “stressors” involved in the normal life history of this animal. Indeed, the reported catastrophic summer mass mortality events are not due to a single factor, but are the result of complex interactions involving environmental parameters, animal physiological condition, and pathogens (Huvet et al. 2004; Li et al. 2007; Malham et al. 2009; Chaney and Gracey 2011). The mean summer (June–August) SST for this region is 21–22°C (Hadley Centre Sea Ice and Sea Surface Temperature data, 01/06/1970 to present [HadiSST]: [http://www.metoffice.gov.uk/hadobs/hadisst/]). Several Hadley Centre coupled atmosphere–ocean climate models (medium greenhouse gas emission scenario, Representative Concentration Pathway 4.5) were examined and all predicted SST for this region to rise between 0.1 and 0.2°C per decade. Hence, within 100 years, the mean summer SST around Faro will likely reach 24°C. Therefore, we need to understand the physiological responses of *C. gigas* and the energetic consequences of cumulative thermal stress during increasingly prolonged chronic intermittent exposure to such high temperatures (Bevelhimer and Bennett 2000). This has consequences not just for *C. gigas* population dynamics but also the biodiversity of species inhabiting the complex intertidal reef structures constructed by *C. gigas*.

## Summary

These data represent one of the first transcriptional profiling experiments using next-generation sequencing on an invasive species, the Pacific oyster *Crassostrea gigas,* and uniquely link these data to physiological analyses into chronic environmental manipulation. Physiological analyses showed that temperature had more impact on adult oysters than pH. Whether this relationship holds for other life-history stages has yet to be determined, but these data show the importance of longer term experiments. Our data show that longer term molecular responses cannot be predicted from short-term acute studies and emphasize the effectiveness of transcriptional profiling as a molecular tool with the identification of novel pathways such as PI3K/AKT/mTOR, which can be investigated further for use in environmental monitoring studies. The advent of cheap sequencing has produced a sea change in our abilities to understand cellular responses in detail in environmental models, given that many of these transcripts are either poorly annotated or such data are absent; the next great challenge will be deciphering the function and interactions of the “unknown” genes.

## Supporting Information

Aditional Supporting Information may be found in the online version of this article:

**Table S1.** Primers used for Q-PCR of candidate genes, including the Accession number from which the primers were designed, putative function of the gene, *R*^2^ and efficiency of the primers.

**Table S2.** Contigs generated in this project from public EST data and used for the SOLiD mapping.

**Table S3.** Annotation of contigs using Blast sequence similarity searching, comparing those transcripts up-regulated in animals cultured at 19 and 24°C under ambient pH conditions.

**Table S4.** Annotation of contigs using Blast sequence similarity searching, comparing those transcripts up-regulated in animals cultured at 19 and 24°C under low pH conditions.

**Table S5.** Annotation of contigs using Blast sequence similarity searching, comparing those transcripts up-regulated in animals cultured at 24°C under ambient pH and 24°C under low pH conditions.

**Table S6.** Annotation of contigs using Blast sequence similarity searching, comparing those transcripts up-regulated in animals cultured at 19°C under ambient pH and 19°C under low pH conditions.
