# Supplementary material for: Identification of molecular and physiological responses to chronic environmental challenge in an invasive species: the Pacific oyster, Crassostrea gigas
Source: Ecol Evol. 2013 Aug 12;3(10):3283–97. doi: 10.1002/ece3.719 (PMC3797477; doi:10.1002/ece3.719)
Supplement: Supplementary file 1 [file ece30003-3283-SD1.doc]

| **Abbreviated**  **name** | **Full name** | **Primers** | | **Accession number** | **Functional indicator** | **R^2^** | **Efficiency** |
| --- | --- | --- | --- | --- | --- | --- | --- |
| Actin | Actin | F  R | GCCCTGGACTTCGAACAA  CGTTGCCAATGGTGATGA | O17320 | Structural: control sequence | 0.992 | 100.34 |
| ATPase | Sodium/Potassium transporting ATPase | F  R | GCTCACATGTGGTTTGATGG  AACATAGCAACACGGGCAAG | B3F730 | Membrane proton transport | 0.983 | 86.13 |
| Hsp70 | Heat shock protein 70kDa | F  R | GAACGACGCCCAGCTACGTC  CTTGCGGCCTATCAGCCTCT | Q75W51 | Protein folding | 0.982 | 86.13 |
| Bone-specific | Similar to bone-specific cmf608 | F  R | CATCGCTGTCTCCGGGTTTC  CCGCGAAAGGAAGATGACGAC | AM685815 | cmf608 is a unique marker of osteochondroprogenitor cells in humans | 0.989 | 67.2 |
| Calreticulin | Calreticulin | F  R | ATTGACTGTGGGGGTGGATA  ACCTTCTTGGTGCCTGGTC | A5LGG9 | Calcium binding protein | 0.984 | 109.0 |
| Protein-Kinase | Calcium dependent protein kinase | F  R | AGCAGCATGGCGGATTTTTC  AGCCGGACTTCTGCAACACA | Q5YIE3 | Calcium binding protein | 0.947 | 100.2 |
| Ferritin | Ferritin | F  R | CCAATGTCGCCAAAATTACC  GGGCCACATCATCTCTATCG | Q2PDG9 | Oxidoreductase | 0.990 | 88.5 |
| Calnexin | Calnexin | F  R | CCTCAAGTCCAAAGCAAAGC  AGGCTCCTCCACAGTCGATA | A5LGG8 | Calcium binding protein | 0.946 | 58.8 |
| GRP78 | Glucose regulated protein 78kDa | F  R | GCCTTCACAGCAGACAATGA  CCGTATCACCCCATCCTATG | Q75W49 | Protein folding | 0.994 | 80.9 |
| Hsc72 | 71kDa heat shock cognate protein | F  R | GAGGATCGCAGCCAAGAA  TATCGCCCTCGCTGATCT | Q9XZJ2 | Protein folding | 0.990 | 88.3 |

**Supplemental Table 1:** Primers used for Q-PCR of candidate genes, including the Accession number from which the primers were designed, putative function of the gene, R^2^ and efficiency of the primers.
