# Supplementary material for: Identification of molecular and physiological responses to chronic environmental challenge in an invasive species: the Pacific oyster, Crassostrea gigas
Source: Ecol Evol. 2013 Aug 12;3(10):3283–97. doi: 10.1002/ece3.719 (PMC3797477; doi:10.1002/ece3.719)
Supplement: Supplementary file 3 [file ece30003-3283-SD3.doc]

| **Contig ID** | **Accession no** | **Gene** | **E value** | **Function** |
| --- | --- | --- | --- | --- |
|  | | | | |
| **Up-regulated contigs from animals cultured at 19°C under ambient pH conditions** | | | | |
| 15913 | Q14498 | RNA binding protein 39 | 1e-123 | mRNA metabolism |
| 17743 | Q8BTM8 | Filamin-A | 9e-25 | Cytoskeletal |
| 22009 | Q4V9Y5 | Cysteine rich secretory protein | 4e-25 | Promotes matrix assembly |
| 354 | A6NMZ7 | Collagen alpha-6(VI) chain | 3e-25 | Cytoskeletal |
| 4396 + 6221 | P02637 | Sacroplasmic calcium binding protein | 2e-57; 3e-48 | Cytoskeletal |
| 6229 | Q13126 | 5-methyl-5’thioadenosine phosphorylase | 3e-98 | Amino acid biosynthesis |
| 7523 | Q2VIQ3 | Chromosome associated kinesin | 8e-41 | Cell division |
| 7797 | P86788 | Gigasin-5 | 5e-32 | Organic shell matrix component |
| No annotation | 10623, 10693, 10803, 13976, 14599, 14730, 15299, 17731, 178338, 18942, 19299, 19698, 3523, 5935, 7335, 7401 | | | |
|  | | | | |
| **Up-regulated contigs from animals cultured at 24°C under ambient pH conditions** | | | | |
| 10340 | Q5R4K9 | Protein kinase C iota type | 2e-70 | Protects against apoptosis |
| 10381 | Q8IZQ1 | WD repeat and FYVE domain containing protein | 4e-88 | Cell autophagy |
| 10397 | Q8CHW4 | Translation initiation factor eIF-2B sub-unit | 1e-21 | Translation |
| 10880 | Q13772 | Nuclear receptor coactivator 4 | 2e-17 | Co-activation of peroxisome proliferator receptor (PPAR) |
| 11079 | Q9H2J7 | Sodium-dependent neutral amino acid transporter B(0)AT2 | 2e-35 | Amino acid transporter |
| 11788 | Q8K4Q0 | Regulatory-associated protein of mTOR | 1e-66 | Cell growth, survival/ autophagy |
| 11926 | A0JM12 | Multiple epidermal growth factor-like domains protein | 1e-25 | Cell adhesion and myogenesis |
| 12130 | O43826 | Glucose-6-phosphate translocase | 2e-15 | Glucose control |
| 12935 | P59222 | Scavenger receptor | 9e-28 | Probable adhesion protein |
| 15688 | Q5I0D6 | Nostrin | 2e-11 | Nitric oxide synthase trafficker |
| 15774 | Q66IC8 | Tctex1 domain containing protein | 1e-14 | Molecular motor |
| 17885 | Q99758 | ATP binding cassette family member | 1e-70 | Lipid trafficker |
| 18422 | Q4R779 | Nucleolar protein | 1e-102 | Translation |
| 18546 | Q5RFZ7 | FAM167A protein | 1e-24 | Unknown |
| 1913 | Q6INS1 | F-box/LRR repeat protein | 2e-17 | Protein modification |
| 19410 | Q90X99 | Lysozyme | 4e-15 | Antimicrobial |
| 19680 | Q13867 | Bleomycin hydrolase | 1e-120 | Protease |
| 19940 | P90893 | Serine protease | 5e-37 | Protease |
| 20328 | K1QM85 | StAR-related lipid transfer protein 3 | 6e-62 | Lipid metabolism |
| 20938 | P81628 | Endoplasmic reticulum resident protein | 3e-33 | Potential chaperone |
| 2217 | F1M8L5 | Propionyl-CoA carboxylase | 1e-37 | Oxidation of fatty acids |
| 3170 | Q96JN8 | Neuralised-like protein 4 | 1e-63 | Cell fate decisions |
| 4909 | P25407 | STI-1-like protein | 2e-18 | Protein-protein interactions |
| 4950 + 4951 | Q96QK1 | Vacuolar protein sorting-associated protein | 1e-126; 1e-117 | Protein/lipid trafficking |
| 7313 | Q96MU7 | YTH domain containing protein | 1e-69 | mRNA processing |
| 7546 | Q9C0G0 | Zinc finger protein | 2e-45 | Transcription factor |
| 8864 | Q96MM6 | Heat shock 70kDa protein 12B | 5e-12 | Lipid interactions/ cell adhesion |
| 9214 | Q6GPM1 | Zinc finger RNA-binding protein | 3e-53 | Transcription factor |
| No annotation | 10087, 10656, 11016, 11960, 12023, 12423, 12467, 13213, 13534, 15609, 17208, 17437, 17978, 18200, 18602, 19901, 20050, 21001, 2226, 2527, 2555, 2568, 2864, 3517, 3690, 4101, 4162, 4314, 4481, 4524, 5525, 6213, 7206, 7430, 7534, 7885, 9678 | | | |

**Supplemental Table 3:** Annotation of contigs using Blast sequence similarity searching, comparing those transcripts up-regulated in animals cultured at 19°C and 24°C under ambient pH conditions. This was to illustrate the effect of temperature on oyster metabolism. Contigs are annotated with the accession number, gene name and expect score of the most similar Blast match. The major function of the gene identified by the Blast sequence similarity searching is also given.
