# Supplementary material for: Identification of molecular and physiological responses to chronic environmental challenge in an invasive species: the Pacific oyster, Crassostrea gigas
Source: Ecol Evol. 2013 Aug 12;3(10):3283–97. doi: 10.1002/ece3.719 (PMC3797477; doi:10.1002/ece3.719)
Supplement: Supplementary file 4 [file ece30003-3283-SD4.doc]

| **Contig ID** | **Accession no** | **Gene** | **E value** | **Function** | |
| --- | --- | --- | --- | --- | --- |
|  | | | | | |
| **Up-regulated contigs from animals cultured at 19°C under low pH conditions** | | | | | |
| 11205 | O02485 | Uncharacterised | 2e-63 | | May be involved in cellular differentiation |
| 13188 | P41436 | Apoptosis inhibitor IAP | 6e-32 | | Apoptosis |
| 14731 | Q862Z3 | Uromodulin | 8e-13 | | Receptor, may be involved in osmotic pressure |
| 1602 + 5863 | Q9Y6A5 | Coiled coil domain containing protein | 3e-21 + 3e-64 | | Cell growth and differentiation |
| 16944 | Q5R4R7 | Protein KIAA1045 | 6e-32 | | Zinc finger transcription factor |
| 17182 | K1PX38 | Cysteine rich secretory family member | 1e-125 | | Signalling |
| 18838 | O14463 | Thioredoxin-1 | 9e-26 | | Antioxidant |
| 19170 | K1R7K5 | Kynurenine-oxoglutarate transaminase | 0.0 | | Amino acid biosynthesis |
| 19302 | K1Q801 | Myosin regulatory light chain | 9e-84 | | Cytoskeletal |
| 19400 | Q14185 | Dedicator of cytokinesis protein | 6e-37 | | Cytoskeletal rearrangements |
| 19784 + 5141 + 7193 | P86785 | Gigasin | 0.0 + 4e-29 + 2e-29 | | Shell matrix protein |
| 20012 | K1S6R2 | N-alpha-acetyltransferase 40 | 1e-170 | | Transcription |
| 20097 | Q9H4L7 | SWI/SNF-related matrix-associated actin-dependent regulator of chromatin subfamily A containing DEAD/H box 1 | 1e-50 | | Replication/chromatin modelling |
| 20183 | Q4UMH6 | Putative ankyrin repeat protein | 2e-12 | | Protein-protein interactions |
| 20722 + 6980 | Q26065 | Actin | 4e-35 + 0.0 | | Cytoskeletal |
| 20880 | Q9D0S4 | Neuralised-like protein 2 | 1e-41 | | Myogenesis |
| 20897 | Q13231 | Chitotriosidase | 7e-48 | | Defense protein |
| 21286 | Q8BW74 | Hepatic leukemia factor | 5e-45 | | Transcription factor |
| 2651 | Q0JRZ9 | FCH domain only protein | 2e-22 | | Involved with low density lipoprotein receptor |
| 3355 | O15990 | Arginine kinase | 1e-132 | | Energy production |
| 4965 | Q9GZ70 | Tropomyosin | 1e-59 | | Cytoskeletal |
| 5007 | Q54G05 | Putative leucine rich repeat containing protein | 9e-18 | | Signalling |
| 5206 | P33262 | Cytochrome P450 | 2e-48 | | Oxidation of organic substances/detoxification |
| 5768 | Q7TSS2 | Ubiquitin-containing enzyme E2 | 8e-15 | | Protein degradation |
| 6138 | P27463 | Retinal dehydrogenase | 7e-65 | | Oxidoreductase |
| 6212 | P02637 | Sarcoplasmic calcium binding protein | 3e-48 | | Abundant in mollusc muscle |
| 6280 | P37397 | Calponin | 7e-33 | | Regulation of smooth muscle contraction |
| 8904 | Q9DG02 | Calcium/calmodulin-dependent protein kinase | 1e-131 | | Signalling and calcium homeostasis |
| 9031 | P33244 | Nuclear hormone receptor FTZ-F1 | 6e-51 | | Transcription factor |
| 9266 | A6NMZ7 | Collagen | 4e-29 | | Cytoskeletal |
| 9600 | Q8N394 | Transmembrane and TPR repeat containing protein | 2e-65 | | Unknown |
| No annotation | 11749, 11803, 12079, 12209, 12285, 12405, 14145, 15324, 15806, 15856, 16026, 16366, 16770, 16919, 17059, 17169, 17418, 17447, 17723, 17731, 17870, 17893, 18342, 18481, 18870, 19097, 19310, 19329, 19413, 19464, 19669, 19835, 19924, 20157, 20716, 21025, 21427, 21657, 22074, 22095, 22110, 2494, 2557, 2745, 3981, 4074, 4164, 4303, 4308, 4410, 4425, 4530, 4566, 4596, 4752, 4926, 5135, 5264, 5494, 5699, 5985, 6326, 6544, 6596, 6832, 6901, 7458, 8419, 8654, 9571, 9962 | | | | |
|  | | | | | |
| **Up-regulated contigs from animals cultured at 24°C under low pH conditions** | | | | | |
| 10711 | Q6UWE0 | E3 ubiquitin-protein ligase | 2e-30 | | Protein degradation |
| 10903 | K1R3V2 | Sushi | 1e-155 | | Cell attachment |
| 10942 | Q7Z0T3 | Temptin | 3e-26 | | Pheromone |
| 11204 | Q922R8 | Protein disulfide-isomerase | 2e-87 | | Protein folding |
| 11241 | Q63191 | Apical endosomal glycoprotein | 5e-20 | | Transport |
| 13071 | P56564 | Excitory amino acid transporter | 1e-122 | | Transport |
| 13935 | P10079 | Fibropellin | 4e-51 | | Extracellular matrix component |
| 14020 | Q29RU2 | Oncoprotein-induced transcript | 4e-12 | | Potential secreted protein |
| 16449 | P18426 | S-crystallin | 5e-23 | | Antioxidant |
| 17668 | P25122 | Potassium voltage-gated channel | 2e-16 | | Ion permeability of membranes |
| 1900 | Q62902 | Protein ERGIC-53 | 3e-67 | | Recycling of proteins |
| 26026 | Q6XVN8 | Microtubule-asociated protein | 4e-46 | | Formation of autophagosomal vacuoles |
| 2851 | K1Q596 | 60kDa heat shock protein | 1e-151 | | Protein folding |
| 4360 | Q5VYJ5 | MAM and LDL-receptor class A domain containing protein | 4e-24 | | Lipid homeostasis/cell signalling |
| 6867 | Q9IBG7 | Keilin/chordin-like protein | 4e-22 | | Signalling |
| No annotation | 12757, 13488, 13489, 13848, 15607, 16895, 17191, 17443, 17599, 17778, 17832, 18272, 19507, 21859, 21864, 22028, 22061, 2527, 2802, 5384, 6213, 6749,7327, 7820, 9938 | | | | |

**Supplemental Table 4:** Annotation of contigs using Blast sequence similarity searching, comparing those transcripts up-regulated in animals cultured at 19°C and 24°C under low pH conditions. This was to illustrate the effect of temperature under low pH conditions on oyster metabolism. Contigs are annotated with the accession number, gene name and expect score of the most similar Blast match. The major function of the gene identified by the Blast sequence similarity searching is also given.
