# Supplementary material for: Identification of molecular and physiological responses to chronic environmental challenge in an invasive species: the Pacific oyster, Crassostrea gigas
Source: Ecol Evol. 2013 Aug 12;3(10):3283–97. doi: 10.1002/ece3.719 (PMC3797477; doi:10.1002/ece3.719)
Supplement: Supplementary file 5 [file ece30003-3283-SD5.doc]

| **Contig ID** | **Accession no** | **Gene** | **E value** | **Function** |
| --- | --- | --- | --- | --- |
|  | | | | |
| **Up-regulated contigs from animals cultured at 24°C under ambient pH conditions** | | | | |
| 10882 | Q7KM13 | Hairy/enhancer of split related with YRPW motif | 3e-15 | Transcription factor |
| 13827 | Q5R957 | Chloride intracellular channel protein | 1e-32 | Ion channel |
| 13958 | K1Q791 | Cytochrome p450 | 9e-62 | Potential role in calcium homeostasis |
| 14100 + 9837 | Q8K2J9 | BTB/POZ domain-containing protein | 1e-81 + 2e-38 | Neural and muscle development |
| 14255 | P55088 | Aquaporin-4 | 5e-29 | Water channel |
| 14258 | Q8NFW1 | Collagen | 6e-33 | Cytoskeletal |
| 14486 | Q8TB92 | 3-hydroxymethyl-3-methylglutaryl-CoA lyase | 1e-100 | Catabolism of amino acids |
| 14665 | Q9VZW5 | FMRFamide receptor | 8e-18 | Receptor for FMRF amide peptides |
| 14689 | Q96KQ7 | Histone-lysine N-methyltransferase | 2e-14 | Transcription regulation |
| 14807 | K1Q760 | Phosphoenolpyruvate carboxylase | 0.0 | Carbohydrate biogenesis |
| 14853 | Q9UBI9 | Headcase protein | 4e-76 | Developmental protein |
| 14856 | Q9I9M5 | Frizzled | 7e-48 | Receptor for wnt proteins |
| 16303 | Q810T5 | Histone acetyltransferase | 1e-84 | Transcriptional regulation |
| 16990 | Q9Z1L0 | Phosphatidylinositol-4,5-bisphosphate 3-kinase | 1e-54 | Phosphorylates Ptdlns/cell survival and apoptosis |
| 17041 | O17966 | DNA topoisomerase | 4e-35 | DNA replication |
| 17214 | Q3UMR5 | Coiled coil domain containing protein | 3e-36 | Mitochondrial calcium uniporter |
| 17360 | Q9U943 | Apolipophorin | 8e-48 | Lipid transport |
| 19865 | K1QDC6 | D-2-hydroxyglutarate dehydrogenase | 1e-153 | Oxidoreductase |
| 19940 | P90893 | Serine protease | 5e-37 | Proteolysis |
| 20535 | Q642J5 | RNA binding protein fox-1 | 3e-15 | Regulates alternative splicing |
| 20920 | Q9U6M0 | Evolutionary conserved signalling intermediate in Toll pathway | 3e-45 | Immune function |
| 20983 | K1R3V2 | Sushi | 1e-155 | Cell attachment |
| 21074 | Q8VDK3 | ETS-related transcription factor | 2e-15 | Transcription factor |
| 21278 | P21941 | Cartilage matrix protein | 9e-31 | Matrix protein |
| 21282 | Q2IBC0 | Hepatocyte growth factor receptor | 1e-80 | Multiple functions including cell survival |
| 2753 | Q9UGU5 | HMG domain containing protein | 1e-41 |  |
| 3145 | Q62991 | Sec1 family domain containing protein | 1e-41 |  |
| 5234 | O15439 | Multidrug resistance-associated protein | 9e-71 | Organic anion pump/cellular detoxification |
| 6153 | K1QSB0 | Fructose-6,6-bisphosphate | 0.0 | Carbohydrate metabolism |
| 6640 | P54315 | Pancreatic lipase-related protein | 6e-25 | Lipid metabolism |
| 7201 | Q9VUC6 | Formin-like protein | 5e-95 | cytoskeletal |
| 7488 | P24821 | Tenascin | 7e-13 | Neuronal development |
| 8043 | Q01705 | Neurogenic locus notch homolog protein | 1e-21 | Cell fate development |
| 9146 | Q5ZMH1 | Septin | 1e-63 | Activation of actin cytoskeleton |
| No annotation | 10527, 10855, 11016, 11687, 13172, 13721, 14160, 14253, 14472, 1664, 16730, 16776, 17795, 17870, 17904, 18200, 18882, 20101, 20914, 21196, 2130, 21353, 22036, 22065, 2791, 2834, 3759, 3977, 4105, 4393, 4696, 4971, 5114, 5942, 6220, 6292, 6596, 6712, 6756, 7102, 7430, 7801, 7891, 8556, 9556, 9962 | | | |
|  | | | | |
| **Up-regulated contigs from animals cultured at 24°C under low pH conditions** | | | | |
| 10833 + 8733 | Q7ZT42 | Staphylococcal nuclease domain containing protein | 2e-34 + 9e-59 | RNA binding protein |
| 11584 | P49907 | Selenoprotein | 2e-16 | Antioxidant |
| 11812 + 4923 | Q8R422 | CD109 antigen | 5e-28 + 1e-105 | Anti-protease |
| 12559 | Q8NFT2 | Metalloreductase | 7e-39 | Electron transport |
| 14414 | Q19673 | Tyrosinase-like protein | 4e-16 | Pigment production/Inhibits voltage gated potassium channels |
| 14584 | Q26636 | Cathepsin L | 1e-107 | Degradation of proteins in lysosomes |
| 17743 | Q8BTM8 | Filamin | 9e-25 | Cytoskeleton |
| 6132 + 6858 | P12107 | Collagen | 6e-45 + 3e-29 | Anti-protease |
| 6867 | Q9IBG7 | Kielin/chordin-like protein | 4e-22 | Cytoskeleton |
| 8807 | Q9BX95 | Sphingosine-1-phosphate phosphatase | 6e-27 | Synthesizes phospholipids/degrades lipid signalling molecules |
| 8852 | Q6UXX5 | Inter-alpha-trypsin inhibitor heavy chain | 9e-32 | Protease inhibitor |
| No annotation | 11320, 15607, 17191, 17366, 17443, 17599, 17778, 17821, 17832, 18942, 19299, 19537, 21859, 22061, 3883, 4822, 5384, 6273, 9646 | | | |

**Supplemental Table 5:** Annotation of contigs using Blast sequence similarity searching, comparing those transcripts up-regulated in animals cultured at 24°C under ambient pH and 24°C under low pH conditions. This was to illustrate the effect of pH at relatively high temperatures on oyster metabolism. Contigs are annotated with the accession number, gene name and expect score of the most similar Blast match. The major function of the gene identified by the Blast sequence similarity searching is also given.
