# Supplementary material for: Identification of molecular and physiological responses to chronic environmental challenge in an invasive species: the Pacific oyster, Crassostrea gigas
Source: Ecol Evol. 2013 Aug 12;3(10):3283–97. doi: 10.1002/ece3.719 (PMC3797477; doi:10.1002/ece3.719)
Supplement: Supplementary file 6 [file ece30003-3283-SD6.doc]

| **Contig ID** | **Accession no** | **Gene** | **E value** | **Function** |
| --- | --- | --- | --- | --- |
|  | | | | |
| **Up-regulated contigs from animals cultured at 19°C under ambient pH conditions** | | | | |
| 17840 | Q9DA01 | Eppin | 2e-16 | Inhibits protein degrading enzymes |
| 3478 | P11678 | Eosinophil peroxidase | 1e-100 | Potential immune function |
| 8699 | Q6INU8 | Tetratricopeptide repeat protein | 0.0 | Protein-protein interactions |
| No annotation | 14426, 16888, 17191, 18968, 20433, 3595 | | | |
|  | | | | |
| **Up-regulated contigs from animals cultured at 19°C under low pH conditions** | | | | |
| 10381 | Q8IZQ1 | WD repeat and FYVE domain containing protein | 4e-88 | Autophagy |
| 10949 | Q7ZX75 | LMBR1L protein | 3e-61 | Membrane receptor |
| 11166 | Q28983 | Zonadhesin | 2e-26 | EGF domain protein |
| 11186 | Q9H222 | ATP-binding cassette family member | 3e-22 | Transporter |
| 11587 | A4IGL7 | Peroxidasin | 4e-27 | Antioxidant |
| 11913 | Q9VCA2 | Organic cation transporter protein | 4e-97 | Cation membrane transport |
| 12324 | Q9D2V8 | Major facilitator superfamily domain containing protein | 1e-38 | Transporter |
| 13342 | P16109 | P-selectin | 6e-13 | Immune response |
| 13518 | Q7YT83 | Substrate-specific endopeptidase Tex31 | 6e-45 | Protease |
| 14452 | Q1HG43 | Dual oxidase maturation factor | 2e-26 | Transmembrane protein transport |
| 14493 | Q99PV0 | Pre-mRNA-processing-splicing factor | 0.0 | mRNA maturation |
| 14807 | K1Q760 | Phosphoenolpyruvate carboxylase | 0.0 | Glucose homeostasis |
| 18772 | F6M2H4 | Big defensin | 2e-91 | Immune function |
| 20938 | P81628 | Endoplasmic reticulum resident protein | 3e-33 | Processing secretory proteins |
| 21779 | P61255 | 60s ribosomal protein | 3e-50 | Transcription |
| 6763 | Q8C6U2 | PQ-loop repeat containing protein | 6e-30 | Multipass membrane protein |
| 6771 | A6QP79 | Collectin-12 | 2e-18 | Scavenger protein, host defence |
| 681 | Q4AEH5 | Glutathione peroxidase | 9e-36 | Antioxidant |
| 7546 | Q9C0G0 | Zinc finger protein | 2e-45 | Transcription factor |
| 7626 | Q9HBY0 | NADPH oxidase | 8e-61 | Host defence |
| 8864 | Q96MM6 | Heat shock protein 70kDa 12B | 5e-12 | Protein folding |
| 9992 | P50429 | Arylsulfatase | 5e-33 | Involved in extracellular matrix |
| No annotation | 10737, 10878, 10939, 1109, 11765, 1210, 13165, 13213, 13350, 13453, 13457, 14394, 14478, 14599, 14845, 15786, 16537, 16583, 17208, 17418, 17843, 18093, 18452, 18542, 18942, 19203, 19441, 19669, 21230, 21463, 21770, 2292, 3501, 3673, 3690, 3839, 4101, 4273, 4345, 4926, 5381, 5422, 5599, 6071, 6275, 6377, 6545, 6684, 6726, 6985, 8412, 8775 | | | |

**Supplemental Table 6:** Annotation of contigs using Blast sequence similarity searching, comparing those transcripts up-regulated in animals cultured at 19°C under ambient pH and 19°C under low pH conditions. This was to illustrate the effect of pH at relatively low temperatures on oyster metabolism. Contigs are annotated with the accession number, gene name and expect score of the most similar Blast match. The major function of the gene identified by the Blast sequence similarity searching is also given.
